# Tablet Apps to Support First School Inclusion of Children With Autism Spectrum Disorders (ASD) in Mainstream Classrooms: A Pilot Study

**DOI:** 10.3389/fpsyg.2018.02020

**Published:** 2018-10-23

**Authors:** Charles Fage, Charles Y. Consel, Emilie Balland, Kattalin Etchegoyhen, Anouk Amestoy, Manuel Bouvard, Hélène Sauzéon

**Affiliations:** ^1^Inria Bordeaux – Sud-Ouest Research Centre, Talence, France; ^2^Laboratoire HACS, Handicap, Activité, Cognition et Système Nerveux, Université de Bordeaux, Bordeaux, France; ^3^Centre Ressources Autisme, Centre Hospitalier Charles Perrens, Bordeaux, France

**Keywords:** autism spectrum disorders (ASD), school inclusion, assistive technologies for persons with disabilities, intellectual disability, adaptive behavior

## Abstract

The inclusion of children with autism spectrum disorders (ASD) in mainstream classrooms is dramatically impeded by their difficulties in socio-adaptive behaviors. This paper presents a package of mobile applications consisting of both assistive and cognitive rehabilitation applications to support first school inclusion of children with ASD. These applications have been tested in a 3-month intervention in mainstream schools and at home, involving 50 participants (30 children with ASD, half of which was equipped and 20 equipped children with intellectual deficiencies). Benefits on socio-adaptive behaviors and social response in school settings, and socio-cognitive functioning have been assessed. The main results showed that equipped children with ASD improved their socio-adaptive behaviors and their social-response in school settings. Both equipped groups increased their socio-cognitive functioning.

## Introduction

Mobile health, the use of mobile digital technologies to improve health care, is a rapidly expanding area, particularly within psychological care of neurodevelopmental disorders such as autism spectrum disorders (ASD) or intellectual disabilities (ID) (Mechling, 2007). The number of mobile applications targeting cognitive training or activity assistance for all kind of disabilities grows up on digital applications stores, such as Apple Store (IOS) or Google Play Store (Android) (Donker et al., 2013). The use of mobile devices in everyday life situations offers new possibilities in terms of rehabilitation, assistance and evaluation, so far impossible due to required presence of a trained stakeholder. Up to 300 applications for children with ASD are now inventoried in applications stores. The appetence of children with ASD for this type of interactive mobile supports (for review: Stephenson and Limbrick, 2013) has undoubtedly been a driver for the expansion of this market. Unfortunately, there is a paucity of controlled studies investigating the effectiveness of psychological interventions based on mobile apps (Stephenson and Limbrick, 2013). The purpose of the present pilot study was to develop and to assess the efficacy of both a set of remediation applications for socio-cognitive functioning and a set of assistive applications for socio-adaptive behaviors in the ecological situation of a first inclusion of children with ASD in mainstream classroom.

### ASD and School Inclusion

The variety of cognitive profiles among children with ASD urges for individual cares and personalized assistance. This will result in overpassing the barriers of their social participation, which take the form of normalized expectations imposed by mainstream social environments such as schools (Charman, 2004; Van Hees et al., 2015). Yet, there is growing evidence that educational inclusion produces a positive effect on children with ASD, especially in terms of outcomes in schooling duration and occupational future (Hunt and McDonnell, 2007). Despite such positive outcomes, inclusive education of these children is often hampered by the misgivings of school staff that presumes negative outcomes on classroom functioning if the student is not autonomous enough (Harrower and Dunlap, 2001). This restriction in social participation in mainstream school settings is often explained by limitations in socio-adaptive capabilities, mainly related to impairments in socio-cognitive functioning of children with ASD (Jackson, 2008). Indeed, when these specific needs are not addressed, they often result in interruptions during class that decrease learning opportunities, not only for the child with ASD, but also for all the students (McCurdy and Cole, 2013). Therefore, supporting socio-adaptive capabilities, as well as socio-cognitive functioning, is a critical need for successful school inclusion of children with ASD.

Leveraging research on Autism, the International Classification of Functioning, Disability and Health, Children and Youth version (ICF-CY, World Health Organization [WHO], 2002) draws up a list of the main domains of needs that are specific for children with ASD and are mostly responsible for the situation of school disability. This list includes social skills, emotion recognition, emotion self-regulation, and executive functioning. As such, children with ASD exhibit variable verbal interaction limitations (Rapin and Dunn, 2003) and difficulties to perform numerous social protocols required for mainstream environments (greeting, thanking, etc.) (Jahromi et al., 2013). Related to their socio-adaptive difficulties, children with ASD exhibit impairments on socio-cognitive processing, and notably the executive functioning-based ability to adapt one’s affective or behavioral response to socio-environmental contexts (Jahromi et al., 2013). These impairments lead to limited ability to recognize and label emotions, which appears to be predictive for social behaviors and academic success among children at risk (Izard et al., 2001); they also lead to disrupting social interactions with peers and their teachers (Peterson et al., 2009). All these impairments are related to failures in emotion self-regulation, such as exacerbated emotional responsiveness, which are common in children with ASD and known to impede their school inclusion (Jahromi et al., 2013). Other impairments are common among children with ASD. They can exhibit executive functioning disorders (activity planning, time management, inhibition, flexibility) such as context-appropriateness of use of knowledge, highlighted in the “executive dysfunction account” by Ozonoff et al. (1991), or perceptual processing disorders as described in the “enhanced perceptual functioning” hypothesis (Mottron et al., 2006). Learning disabilities (as described in theory of “enhanced discrimination and reduced generalization,” Plaisted, 2001, p. 10) can also be found among children with ASD. Taken together or separately, these cognitive disorders result in difficulties in maintaining attention, listening and mimicking. Such limitations are challenging for classical instructional procedures (Charman, 2004; Van Hees et al., 2015).

### Literature Review: Cognitive Interventions for Children With ASD

There are two major approaches of cognitive interventions that are commonly used for children with ASD.

The first approach, called Cognitive Behavioral Therapy [CBT; e.g., *Applied Behavior Analysis* - ABA, Reichow, 2012; *Treatment and Education of Autistic and related Communication handicapped Children* - TEACCH, Ospina et al., 2008 (Ho et al., 2015)], is skill-oriented and focuses on adaptive behaviors reinforced by rewards (for review: Ospina et al., 2008; Ho et al., 2015). This individualized program relies on a rigorous time and space structuring of learning through paper-based visual supports, and a close collaboration between the education staff and family members (Panerai et al., 2002).

The second approach gathers cognitive process-oriented interventions, focusing on the cognitive capabilities underpinning the task performance (for example, Tanaka et al., 2010). We name such interventions *Cognitive Remediation Intervention* (CRI). Importantly, the two approaches are not orthogonal, but rather appear to address interrelated cognitive components to an integrated whole. Thus, they should ideally be used together into a multidimensional intervention for significant improvements of socio-adaptive behaviors communication and, cognitive functioning of children with ASD (Ozonoff et al., 1991; Solomon et al., 2004; Stichter et al., 2012).

A main limitation of CBT studies as well as CRI studies is their weak power of ecological validity since they rely on work and evaluations both conducted on specialized environments (i.e., therapist’s office). Thus, transferring therapeutic learning in mainstream environments is rarely evaluated, or partially reported (Ospina et al., 2008; Ho et al., 2015). Consequently, objective data remain lacking to determine whether these interventions can improve adaptive abilities and socio-cognitive functioning of children with ASD in daily life. Probably, the barriers related to real-life settings participate to explain this weakness of ground truth of both CBT and CRI based studies.

Importantly, these aforementioned interventions are currently embedded into psycho-educative tools to facilitate mainstream school inclusion for children with ASD. For instance, the Picture Exchange Communication System used in CBT supports children during communication activities through paper-based picture folders (Carr and Felce, 2007). As well, sequencing activities on illustrated sub-tasks help children organize and manage their time (McClannahan and Krantz, 1999). Similarly, related to CRI, social stories are commonly used to train children to cope with typical social situations (Karkhaneh et al., 2010). Although the effectiveness of these methods has been reported, they have important drawbacks. They are time-consuming (e.g., activity schedules, Hayes et al., 2010), stigmatizing (e.g., cumbersome material of paper-based folders), and are thus not adapted to the pervasive needs of these children across various contexts of mainstream inclusion (e.g., social stories) (Fage et al., 2014).

### *Mobile* Technology-Based Interventions (*m*TBI) for ASD

Since 2010, touch-screen tablets have now become commonly used in school settings. They support new paradigms for teaching, provide tools for individual or collective work, and target a large variety of activities. Despite a growing number of applications for children with ASD, there is little evidence of their efficacy in terms of compensatory or therapeutic effects in everyday functioning as well as cognitive functioning of children using such apps (Ramdoss et al., 2011; Ploog et al., 2013; Grynszpan et al., 2014). These *m*TBI consist of either training tools for challenging tasks or assistive applications to help performing activities. Ploog et al. (2013) proposed a classification of technologies for ASD into four main domains, i.e., (1) language (e.g., Bernard-Opitz et al., 1999), (2) social skills (e.g., Nikopoulos and Keenan, 2007), (3) emotion recognition (e.g., Silver and Oakes, 2001), and (4) socio-cognitive processing (e.g., Swettenham, 1996). On the latter domain, the *Mind Reading* software is often referenced as a gold standard because of both its large number of exercises proposed on emotion identification and socio-cognitive mechanisms, and its experimental validation (Golan and Baron-Cohen, 2006). Indeed, benefits on trained processes are reported in studies with strong experimental design standards (number of participants, standardized measures, etc.). However, these benefits are not documented in real life setting.

For the three other domains of applications, experimental design standards, such as sufficient number of participants or recruitment of a control group, are rarely reached (for review: Ploog et al., 2013). Additionally, the training transfer toward real situations is usually not observed, when evaluated (for review: Ploog et al., 2013).

Aside from the cognitive remediation applications, a large number of technologies addresses *in situ* support, providing assistance when children with ASD are actually performing tasks (for review: Mechling, 2007). Such compensatory technologies largely rely on activity schedules, which divide activities in sequences of steps depicted by a written statement and a picture (McClannahan and Krantz, 1999). Their efficacy to assist extra-curricular activities of children (e.g., hand washing, waiting time during medical visits, etc.) has been reported through several studies (Mechling, 2007; Ben-Avie et al., 2014). We believe that such efficacy may result from a compensatory assistance to executive difficulties associated with ASD and/or to their exacerbated anxiety (i.e., explicit nature and invariant structure of application interface, Hayes et al., 2010). As promising as they may be, these studies involved very few participants (from 1 to 10, sometimes without control counterparts) and usually took place in specialized environments (e.g., special-education classrooms, specialized agencies, etc.) and aimed more toward demonstrating the usability of the technologies for children with ASD, rather than demonstrating their clinical efficacy (for review: Grynszpan et al., 2014). Therefore, albeit activity schedules embedded on *m*TBI yielded promising results in specialized environments, their efficacy to assist tasks in mainstream environments is still to be investigated in children with ASD.

Regarding mainstream school settings specifically, relatively few devices based on digital systems have been developed to support inclusion. For example, MOSOCO is a smartphone-based tool to practice social skills during school breaks, by using an augmented reality approach (Escobedo et al., 2012). Three children with ASD and nine matched typically developing control children used MOSOCO. Authors reported increased number of interactions between participants with ASD and typically developing children as well as increased interaction duration for children with ASD while reducing their interaction missteps. The vSked system is an application for creating and managing pictured activity schedules and destined to children with ASD. This application was designed with respect to interviews (families, teachers, therapists, special-education teachers, and neuroscientists) and direct observations in three special-education classrooms (Hirano et al., 2010). A special-education classroom including nine children with ASD was equipped with vSked. Qualitative results in terms of reducing education staff burden when using visual supports and improving communication and social interaction between children are reported. For another example, a task manager, implemented on smartphone, has been used by young adults with ASD at university (Gentry et al., 2010). Twenty-two high-school students were equipped with the PDA-based task manager. At the end of the 8-week intervention, participants exhibited increased occupational performance, as well as autonomous use of the assistive tool. Lastly, the ICan application, a digital and configurable version of PECS, has been tested in special-education classrooms with teachers including children with ASD, to assess its usability (Chien et al., 2015). Eleven children with ASD used ICan during 4 weeks. Like Hirano et al. (2010), the authors reported reduced burden for caregivers through reduced time spent on preparing visual support. They also reported enhanced “willingness to learn and interact with others” for equipped children with ASD. These first studies can be seen as a first step toward mobile TBI (*m*TBI) in mainstream classroom for the inclusion of children with ASD by showing the feasibility of introducing *m*TBI in the school environment. However, their clinical efficacy remains to be investigated since these studies do not provide any empirical support of gains for socio-adaptive behaviors in school settings, or even for socio-cognitive functioning.

### School Context: Including Students With Intellectual Disabilities (ID)

Special-education classroom often gathers students with various conditions; they are mostly students with ASD and students with non-specific ID (Duncan and Murnane, 2014). When introducing an assistive technology in special-education classroom, researchers often include both populations in their field studies. For example, Mechling (2007) proposed a literature review of assistive technologies for students with ID, which also included students with ASD. Similarly to students with ASD, students with ID exhibit limited cognitive functioning (i.e., IQ < 70) as well as limited socio-adaptive behaviors (DSM-IV, American Psychiatric Association [APA], 2013), resulting in very limited social participation (Mouga et al., 2015). However, authors reported that socio-adaptive behaviors were more limited with ASD population compared with ID population.

Mobile assistive technologies offer opportunities to support individuals with ID in their daily life. Specifically, such tools mainly support communication and daily life activities. New technologies allowed classical paper-based pictograms used to communicate basic needs to be enriched with synthetic voices (e.g., MyVoice application, Campigotto et al., 2013); such devices are referred to as Speech-Generating Devices (SGD). A recent meta-analysis reported the relevance of SGD-based intervention to improve communication of individuals with ID (Ganz et al., 2017). This population can also benefit from sequencing activities into successive illustrated steps (for review: Koyama and Wang, 2011). Previous reviews revealed promising results for individuals with ID to benefit from interventions based on handheld devices to improve their communication and daily life skills (Kagohara et al., 2013; Stephenson and Limbrick, 2013).

### Aim of the Study

Based on the aforementioned data, we developed a package of apps on mobile tablets to promote the first school inclusion of children with ASD in secondary school settings. This package, named “School+,” consists on both assistive apps (compensatory purpose) and cognitive training apps (remediation purpose) designed with respect to specific existing accessibility principles for children with ASD. Assistive apps consist of two applications implementing activity schedules for verbal and school routines and a self-regulation emotion. Training apps are oriented toward socio-cognitive processes (emotion recognition and attention orientation). In the context of the first inclusion in mainstream classrooms, “School+” *m*TBI was deployed for a 3-month use. The aim of this study is (1) to demonstrate the relevance of tablet-based assistive and training apps to support students with ASD within mainstream classrooms and (2) to demonstrate that students with other conditions (e.g., with ID) who share functional and cognitive limitations can also benefit from using such applications in mainstream classrooms. We hypothesized that the deployment of such applications would lead to (1) improvements of socio-adaptive behaviors (school disability scale, EQCA-VS, Morin and Maurice, 2001) greater for students with ASD compared with students with ID (as they exhibit slightly better socio-adaptive behaviors, (2) improvements of social skills [Social Responsiveness Scale (SRS), Constantino et al., 2003] for students with ASD, and (3) improvements in socio-cognitive functioning (cognitive evaluations related to socio-cognitive mechanisms) for both equipped groups (i.e., students with ASD and students with ID).

## Materials and Methods

In order to measure benefits related to uses of both assistive and remediation applications, three groups of children were recruited. Two of these groups were composed of children with ASD: one group was equipped with the applications (equipped ASD), one was not (non-equipped ASD). An equipped group of children with ASD was compared with a non-equipped group of children with ASD to capture intervention effect for children with ASD. The third group, recruited in the same special-education classrooms, was composed with children with Intellectual Disability (equipped ID). An equipped group of children with ASD was compared with an equipped group of children with ID to evaluate specific and shared effects of our intervention across populations with different medical conditions (i.e., using a cross-syndrome method^1^, Sigman and Ruskin, 1999).

### Participants

Our study took place in French public secondary schools of the Bordeaux agglomeration, where special-education classrooms are implemented for fostering school inclusion opportunities (Fage et al., 2017). A total of 50 students aged from 12 to 17 years were recruited. Two equipped participants moved to another curriculum or a specialized institution before the end of the 3-month intervention; they were removed from the study. Finally, 29 of our participants were students with ASD and 19 others were students with ID (see Table 1). Students with ASD were separated into two groups: 14 equipped children (tablet-ASD) and 15 non-equipped control children (control-ASD. The three groups were matched with the chronological age, the intellectual functioning (according to the IQs estimated from abbreviated WISC-IV (Grégoire, 2000), and on performance on three socio-cognitive tests. These tests included Picture Sequencing test (intentions detection; Baron-Cohen et al., 1986), Look in My Eyes test (emotion recognition through a sight; Baron-Cohen et al., 1997), and a Dynamic Emotion Recognition test (emotion recognition through videos; Tardif et al., 2007). Possible differences between the three groups of children were tested using a one-way analysis of variance (ANOVA) (Table 1). Neuro-pediatricians examined all the children and the ASD diagnosis was made according to the criteria of the DSM-IV (American Psychiatric Association [APA], 2013) and with respect to the “Autism Diagnostic Interview-Revised” scale (Lord et al., 1994). Note that researchers were single-blinded: they did not know the medical condition of the participants during the experiment. Indeed, groups were formed *a posteriori* at the end of the intervention, when neuro-pediatricians provided the medical diagnoses.

**Table 1:** Participant’s characteristics.

	Tablet-ASD (*N* = 14) (males: 14; females: 0)	Tablet-ID (*N* = 19) (males: 9; females: 10)	Control-ASD (*N* = 15) (males: 13; females: 2)	*p*-Value
Age	14.26	14.23	14.16	
*(SD)*	(0.26)	(0.29)	(0.43)	0.977
IQ	69.07	60.53	71.13	
*(SD)*	(8.19)	(4.50)	(8.51)	0.495
Picture sequencing	8.07	8.47	8.33	
*(SD)*	(1.89)	(0.84)	(1.13)	0.962
Look in my eyes	7.57	7.00	8.20	
*(SD)*	(0.86)	(0.76)	(0.96)	0.517
Dynamic emotions recognition	13.07	13.79	11.53	
*(SD)*	(1.26)	(0.81)	(1.66)	0.895

As recommended by the Helsinki convention, teachers and parental written informed consent as well as children’s assent were obtained before participation. Also, the ethics committee affiliated to the University of Bordeaux (Comité de Protection des Personnes) approved the experimental protocol, prior to recruiting participants. Finally, the collecting and processing of digital data have been checked by the COERLE, which is the ethical committee of Inria (National research center in computer sciences) for an official declaration to CNIL (Conseil National Information et Liberté, i.e., National Council of Information and Freedom).

#### Material

Thanks to a previous co-design work with all stakeholders (families, school staffs, therapists), two sets of three apps were developed: (1) *assistive applications* destined to be used inside mainstream classrooms (whenever necessary), and (2) *remediation applications* destined to be used at home on a daily basis (15 min per day visualized by a time-line on screen, five times a week). All contents of these applications specifically aimed school settings. Moreover, they were personalized to each student. Our applications run on a touchscreen tablet (Apple iPad© second generation).

#### Interface Design

**FIGURE 1:**
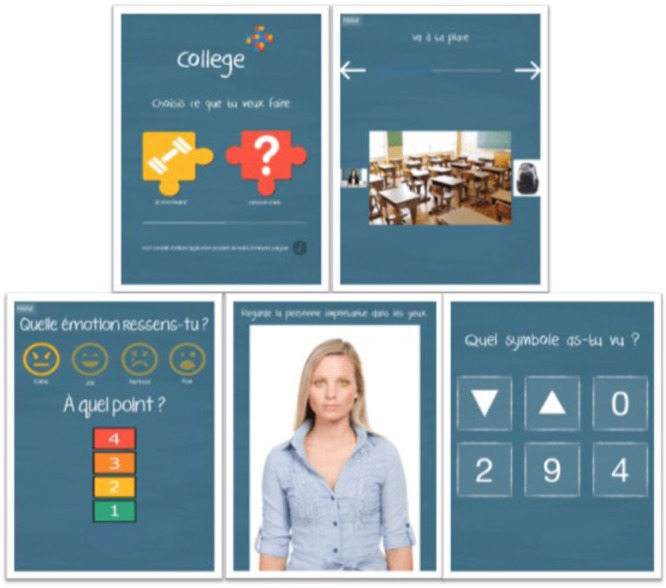
School+ package (“Make your choice”; “Training”; “Assistance”).

Previous human–computer interaction studies identified relevant design principles to ensure the accessibility and usability of technologies by children with ASD. Especially, technological supports need to rely on visual supports, to prevent mistakes, to avoid distractive stimuli, to focus on predictability or display clear mapping between actions and feedback provided by interface (Hayes et al., 2010; Hirano et al., 2010; Porayska-Pomsta et al., 2012; Hourcade et al., 2013). We designed all applications in the “School+” package following these proven guidelines, promoting flexibility of all their contents to match specific needs of each child (Hayes et al., 2010). To complement these guidelines, we extracted design principles from CBT. Notably, we focused on a strong structuring of spaces and times on each screen, as proposed by TEACCH program (Panerai et al., 2002). The same interface was used for two assistive applications using activity schedules. It was also used across the three cognitive remediation applications; same steps were implemented across assistive applications (selection of the appropriate activity schedule, following steps of the sequence) and across cognitive remediation applications (selection of the game, display of the material, input of the user, feedback from the application, prompting to do another exercise or exit). Moreover, as suggested by dedicated approaches such as ABA (Reichow, 2012) and implemented by Lovaas (1987) for example, each application of the “School+” package is dedicated to one specific task, addressing a specific need. As a result, we decided to split assistive applications into two separate applications, both relying on activity schedules sharing the exact same interface: one dedicated to classroom routines and one dedicated to verbal communication routines (see Figure 1).

Regarding cognitive remediation apps, similar design principles were followed. Importantly, rewards, in the form of congratulation messages, have been extensively used across these apps, as advised in ABA and TEACCH. Moreover, a progression bar was displayed at the beginning of each training session for the user to exhibit the time to be spent on exercises on the current day. Finally, stakeholders (i.e., teachers, parents, school aids) were able to keep track of participants’ progresses through a dedicated module, hidden to children.

#### Assistive Applications: Support for Activity Planning–Execution in School Settings

To address the difficulties of planning and executing new tasks faced by children with ASD, two applications are dedicated to training children to perform classroom routines and verbal communication routines in mainstream classrooms. These activities have been identified and validated after interviews with teachers and school staff involved in this study (Fage et al., 2016). Note that for these two applications, the number of activity schedules generated by the users is not limited.

##### Routine app.

The first app addressing with classroom routines targeted following activities: Going to the classroom, Entering the classroom, Taking out school supplies, Taking notes, and Leaving the classroom.

##### Communication app.

The second app focused on verbal communication routines in the context of the classroom. Two communication contexts (i.e., initiation or reception) and two interlocutors (teacher or student) have been distinguished, leading to four types of interaction scenarios. For each scenario, different sequences are proposed based on the goal of the communication (e.g., ask for help, make a comment, ask for repetition).

These two applications presented the same interface (see Figure 1): a list of available sequences is displayed on the first screen; two arrows allow moving forward and backward through sequence steps. A progression bar, plus thumbnails of the previous and the next steps, eased the user to situate in the sequence. Each step is described with a text and picture to reinforce the understanding of the task. Finally, a positive reinforcement message is displayed at the end of each sequence.

##### Emotion regulation app.

The third app proposes assistance for emotion regulation. Firstly, the child is invited to identify its emotion thanks to a set of emoticons related to the four basic emotions: joy, fear, anger, and sadness. The child has then to rate the intensity level of its emotion thanks to a 4-level thermometer (see Figure 1). The child is presented with idiosyncratic soothing contents created with families. Each intensity level is associated with a medium type: soothing statements, co-regulation personal pictures (around 10 pictures selected by each child and their parents) and videos (2–3 min of soothing personal video).

#### Socio-Cognitive Remediation Applications

Based on “serious games” principles, three apps dedicated to socio-cognitive processes were built with an increasing difficulty. Each app provided two levels of exercises moving to the next level when a threshold of 80% successful trials was reached.

Two of these three apps consist on facial emotion recognition exercises: one based on static content (i.e., photos), the other one based on dynamic content (i.e., videos). The third app proposes exercises of visual attention orientation in social situations.

##### Static emotion recognition app.

The child is presented with four photos depicting different facial emotions and asked to identify an emotion given in an instruction, coupled with the corresponding emoticon. The first level includes normed photos of 67 unknown people mimicking seven basic emotions (i.e., joy, fear, anger, sadness, surprise, disgust, and neutral). The second level includes photos of facial expressions of members of the school staff of each participant (special-education teachers, school aides, technical staff, etc.). Each school staff member was told to mimic the same seven basic emotions. The photos of the 20 staff members have been collected for each participant (i.e., 20 different joy photos, 20 different photos of fear, etc.). The application randomly displays one of the seven emotions while ensuring an equal number of occurrences of each emotion in each set.

##### Dynamic emotion recognition app.

The child is presented with the playback of a video. At some point, the video flow is interrupted and a facial recognition task is displayed. The child is asked to select the appropriate emotion on a list of word-emoticon pairs, according to the displayed picture (i.e., the frame displayed when the video stopped). Each video stops at least twice, with a different emotion each time. Emotions are equally represented among each difficulty level. The first level involves videos of simple cartoons with only one character moving in a stripped-down environment (i.e., single color background) and only four basic emotions (i.e., joy, fear, anger, sadness). The second level involves videos of more complex cartoons, textured, with interactions between the characters and the enriched emotions.

Note that the playback of these videos is slowed down on early levels to ease the identification of facial emotions by the children. This slowing down leverages the works revealing the benefits of a slowed-down exposure to dynamic stimuli with an emotional valence for children with ASD (Gepner et al., 2001; Tardif et al., 2007). The audio has been removed from these videos to account for research results stating that sensory multichannel should be avoided when training children with ASD (Mottron et al., 2006).

##### Gaze-orientation app.

This third app (see Figure 1) proposed visual attention focus training. Such skill are documented as participating to the detection of communicational intentions (Kampe et al., 2003), and being impaired among children with ASD (Charman et al., 2000). A photo of a face is presented to the student, and then a symbol is briefly displayed on the eyes of the pictured face. The child is finally asked to select the previously displayed symbol among a list of other symbols. The first level of this application is composed of face photos; the second level is composed of complex interaction pictures in school settings. In this case, the symbol is displayed on the whole face of the relevant person on the given school setting scene (lecture to the whole classroom by the teacher, talk given by a student, chat with a classmate, etc.). For each intensity level, at first, the symbol is displayed for 4 s. Then it is displayed for only 2 s in order to accelerate attention focus orientation.

A feature of training performance monitoring has also been implemented. Thus, any parent or specialized teacher can follow the use of the applications, as well as daily progress of a child.

#### Application Usage Procedures

##### Assistive apps procedure

Children were told to use assistive apps in classrooms, whenever they felt it was necessary. Teachers were told that this situation could occur during their class; they were even encouraged to refer to the tablet when addressing socio-adaptive behaviors. For each child, the school aid was trained to use the School+ apps and received specific instructions to support their use by the child. The school aide was told to redirect the child to the assistive apps, or even to launch the appropriate assistive app herself, whenever she felt the accompanied child could benefit from a visual support.

At the end of each month of intervention, the school aide was asked to indicate whether the child used the application in full autonomy and in adequate manner (scored 1), or whether they had needed help to use it (scored 0) in appropriate situations (e.g., emotional outbursts). The experiment showed that the child independent usage increased from first to third month of intervention for all children [*F*(1,31) = 60.13; *p* < 0.001; η^2^ = 0.660].

##### Training apps procedure

The children were told to use training apps equally for 15 min per day, at least 5 days per week. A progression bar was displayed on the interface to indicate the time spent on training apps and the time left for the current day. Parents were encouraged to accompany their child to use training apps, by checking the time spent on apps and progress made through the performance-monitoring feature.

The parents’ perception was assessed with the USE questionnaire the two items (Lund, 2001) assessing the parent’s perceptions in terms of usability and ease of learning (with a Likert scale from 0 to 4), with a maximum score of 4. High scores of usability occurred among the parents and there was no significant difference between the group of children with ASD (*m* = 3.71; *SE* = 0.13) and the group of children with ID (*m* = 3.74; *SE* = 0.10) [*t*(31) = -0.139; *p* > 0.800]. We report the same results for the ease of use of our application: there was no significant difference between the group of children with ASD (*m* = 3.57; *SE* = 0.29) and the group of children with ID (*m* = 3.63; *SE* = 0.22) [*t*(31) = -0.168; *p* > 0.800]. Thus, apps were perceived as usable and easy to learn by all parents, irrespective of group condition.

##### Application usage verification

In order to objectively assess the application usage by children in mainstream classrooms (for assistive applications) and at home (for training applications), interaction data were recorded for each use of each application. These data provide us with the number of uses of “School+” apps by each participant, during the intervention. Specifically, we collected a number of uses of assistive apps (activity schedules for classroom routines and verbal communication, and emotion-regulation) and a number of uses of socio-cognitive remediation apps (attention orientation, and static and dynamic emotion recognition) for each participant. At the end of the intervention, we compared the number of uses of applications from both equipped groups. We conducted a Student *t*-test comparison with one inter-individual factor with two modalities (equipped ASD and equipped ID). This revealed no statistical differences between groups on assistive application uses [*t*(31) = -0.22; *p* > 0.800] nor on socio-cognitive rehabilitation applications [*t*(31) = 0.40; *p* > 0.400]. Such result ensures that both equipped groups equally used “School+” apps during the intervention. In other words, all participants followed the instructions regarding apps usage in mainstream classrooms and at home.

### Procedure

**FIGURE 2:**
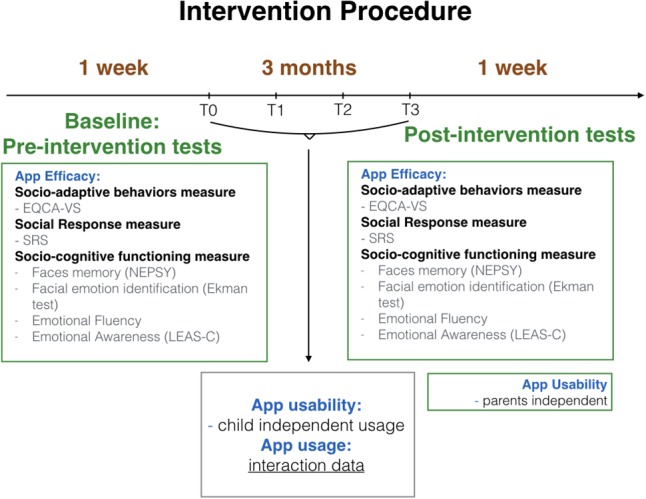
Intervention procedure.

Prior to our intervention, we held a meeting with the inclusion teachers, the special education teacher, the school aide, the parents, and the children. The goal was to give them an overview of our procedures (see Figure 2), to explain the importance of using our application on a regular basis in a synergistic manner, and to answer all their questions. We also gave a demonstration of our tool, explaining its functioning.

Later, we met again with families to create/identify idiosyncratic media contents to personalize the application. Parents were asked to choose “around ten photos or pictures and a short video that was soothing for their child.” In most cases, a conversation between parents and children spontaneously took place. Chosen photos mostly referred to the child, either on vacation or in an environment where they feel safe (usually their home). Videos were often produced for the purpose of our study, showing children practicing their hobbies.

The participants were then observed during their first inclusion in the mainstream classroom (during French, mathematics, history, geography, or biology classes) for 2 weeks. For the purpose of School+ intervention, each participant attended a new class where new situations could occur. It was a 1-h class that occurred once a week during a period of 3 months. A school aide accompanied each child during inclusion. Each school aide was trained to support students with ASD. In addition, they were explained how to use our applications to play the role of social support for their uses during inclusion. During each inclusion class, for each child, the school aide completed a specific questionnaire to collect the activity observations related to the assistive apps.

#### School+ Assessment: Pre-intervention and Post-intervention Measures

The School+ assessment was conducted according to pre- and post- intervention assessment. Two kinds of measurements have been performed: the former was related to socio-adaptive behaviors in school settings and the later was related to socio-cognitive functioning.

During both the baseline assessment session and post-intervention assessment session, the special-education teachers of the children with ASD and the children with ID completed adaptive behavior scales for school settings (EQCA-VS, Morin and Maurice, 2001) and social skills (French version of SRS, Constantino et al., 2003), based on their observations and their knowledge of the child. All children completed neuropsychological tests related with socio-cognitive mechanisms: emotional word fluency (Greenberg et al., 1995), emotional awareness (LEAS-C, Veirman et al., 2011), immediate face memory (subtest NEPSY, Korkman, 1988), and facial emotion recognition (Ekman test, Eckman, 1972).

All post-intervention measures were completed within 2 weeks after the end of the 3-month intervention. All interviews were conducted at school or at home.

#### Measures of Socio-Cognitive Functioning

A battery of four neuropsychological tests evaluating socio-cognitive processes has been used to assess pre- and post-intervention effect.

##### Immediate faces memory (*Subtest NEPSY*, Korkman, 1988)

This test comprises a sample of 16 photos of normed, non-emotionally connoted child faces. Photos are displayed for 5 s each. Afterward, the faces are displayed another time, accompanied with two unknown faces. The instruction given to the child is as follow: “Look at these three faces. You have previously seen one of these children. Show me the one you saw.” The participant has then to point out the answer. Therefore, a maximum score of 16 can be obtained.

##### Facial emotion identification (*Ekman test*, Eckman, 1972)

This test comprises 30 normed photos of faces exhibiting on of the six basic emotions: joy, anger, fear, sadness, surprise, and disgust. Photos are displayed for 5 s each. The person is asked to point out the correct emotion on a text list afterward. A training showing each emotion one time is performed prior to the evaluation. A maximum score of 30 can be obtained. Two different sets of 30 photos have been used on pre and post-intervention assessment to prevent from learning effects.

##### Emotional word fluency test (Greenberg et al., 1995)

This test assesses an individual ability to identify its own emotional states by measuring its access to an emotional lexicon. To do so, the person is asked to produce all the words designating an emotion as fast as possible (within 2 min). The score is the number of words produced that designate an emotional state.

##### Emotional awareness (*LEAS-C*, Veirman et al., 2011)

This test comprises 12 interpersonal scenarios of daily life (mainly in school settings). Each scenario briefly describes a situation involving two characters. The participant is asked to describe her emotional states in the given hypothetical situation, as well as the emotional states of the other character. This distinction allows extracting two sub-scores of the LEAS-C: self and other’s emotional awareness and emotional awareness. Each scenario is meant to induce one of the four basic emotions (i.e., joy, sadness, anger, fear). Each emotion is shown three times in the test. The complexity of the answer regarding the number and the richness of the formulations is rendered on five levels, from 1 to 5. Levels 1 and 2 are related to formulations that do not describe explicitly or poorly an emotional state (e.g., “It would have hurt.”). Level 3 is related to the direct formulation of the basic emotions (e.g., “I would feel sad.”). Level 4 is related to more complex emotional awareness, with formulations involving more than one emotion (e.g., “I would feel happy but maybe also excited.”). Finally, level 5 is related to formulations involving emotional states considering the other character (e.g., “I would feel sad but also happy for my friend.”). Each scenario is scored from 0 to 5: 0 if no answer or irrelevant answer (e.g., “I would feel that she meant it.”), 1 to 5 regarding the level of the answer. The 12 scenarios are divided into two sets of six scenarios each, covering the four emotions. The first set is used for pre-intervention assessment; the other is used for post-intervention assessment. A maximum score of 30 can then be obtained for each set.

In order to compare intervention effects among all neuropsychological tests, all raw scores have been transformed to standard *z* scores.

#### Measure of Socio-Adaptive Behaviors

In order to measure benefits on socio-adaptive behaviors, two scales were used. The teacher of each special-education classroom completed French versions of the Quebec Adaptive Behavior Scale-School Version (EQCA-VS, Morin and Maurice, 2001) and SRS (Constantino et al., 2003). These scales are particularly well-suited for school settings, given their quantitative nature and their strong link to the observations of the teachers in natural environment.

##### EQCA-VS

This scale measures socio-adaptive behaviors clustered into five categories: Communication (17 items), Social skills (17 items), Autonomy (16 items), School skills (25 items), and Leisure (11 items). Each item describes a behavior that can be observed in the school setting. Scoring is as follows: “0” if behavior is not observed; “1” if behavior is only partially performed, with help or prompted by a caregiver; “2” if the behavior is fully performed. The version for teachers has been chosen for the purpose of this study.

##### SRS

This scale measures limitation of social response in terms of social awareness (8 items), social information processing (cognition, 12 items), reciprocal social communication abilities (22 items), social involvement motivation (11 items), as well as repetitive motor behaviors (12 items). The scale consists on 65 items, referring to a social behavior, scored from “1” “not true” to “4” “almost always true.” Note that this scale measures limitations of social response. Therefore, the higher the SRS score, the more the social response is impaired; a cut-off total score (>59) marks a considered pathological social response.

### Design and Statistical Treatments

To measure the efficacy of the School+ intervention, three mixed MANOVAs have been conducted with two within factors and one between factor according to the studied measures as follows: first, socio-cognitive functioning measures, second an adaptive behavior measures in school context and third, social response measures in school context. For each MANOVA, the between factor was Group, which had three levels (tablet-ASD, control-ASD, tablet-ID). The first within factor was Time, which had two levels (pre- and post-intervention conditions). The second within factor differed according to the studied measures, as follows: first, for the socio-cognitive functioning measure, it referred to four domain’s measures (Emotional Fluency, Emotional Awareness, Immediate Face Memory, and Emotion Identification), and second for the adaptive behaviors measure, it referred to the five domains of EQCA-VS: Communication, Social skills, Autonomy, School skills, and Leisure).

If a significant interaction between group and time is observed, then partial MANOVA (Time ^∗^ domain) per group was performed on the socio-cognitive measures, or those for adaptive behavior. And Time comparisons were then performed on each dimension of measures (i.e., *t*-test procedures).

If an interaction effect was reported between the three factors (Time ^∗^ Group ^∗^ domain), partial analyses (ANOVA[(Group)^∗^(Time)]) were conducted to assess intervention benefits across time with respect to group conditions. If an interaction effect was reported between the two factors (Time ^∗^ Group), comparisons were conducted for each group to capture differential effects of Time factor on each measure (i.e., *t*-test procedures).

**FIGURE 3:**
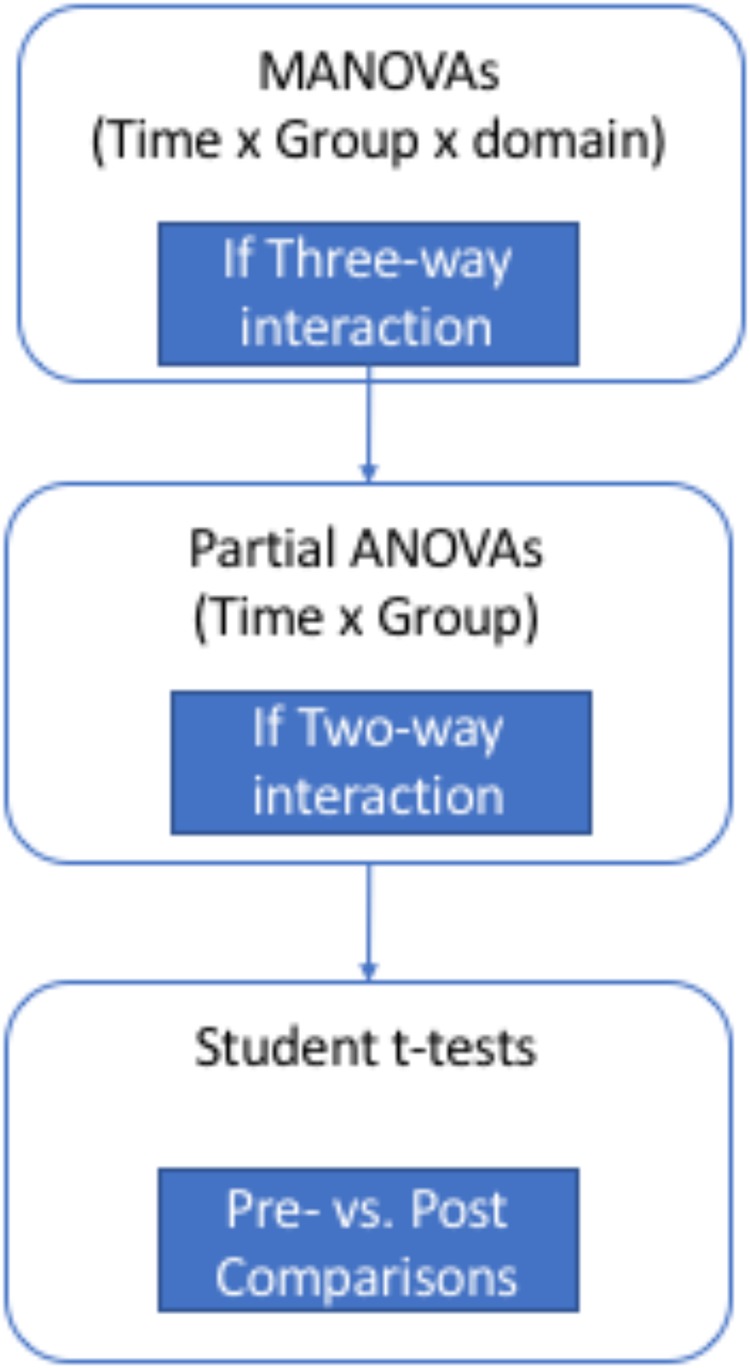
Flowchart of statistical analysis.

All dependent measures were numeric. SPSS-19 software has been used for all statistical analyses. For an overview of the statistical analysis flowchart, see Figure 3.

## Results

For the sake of conciseness, we only report and discuss the significant results in this section. The presentation of means, standard deviations, and entire statistical results for each measure is deferred to the Appendix (Tables 2, 3a, 4, and 4a). The strength of statistical effects (effect size) is reported through η^2^ computed separately from SPSS (which only provides $\eta_{p}^{2}$).

### Socio-Cognitive Functioning (Neuropsychological Tests) (See Figure 4)

**FIGURE 4:**
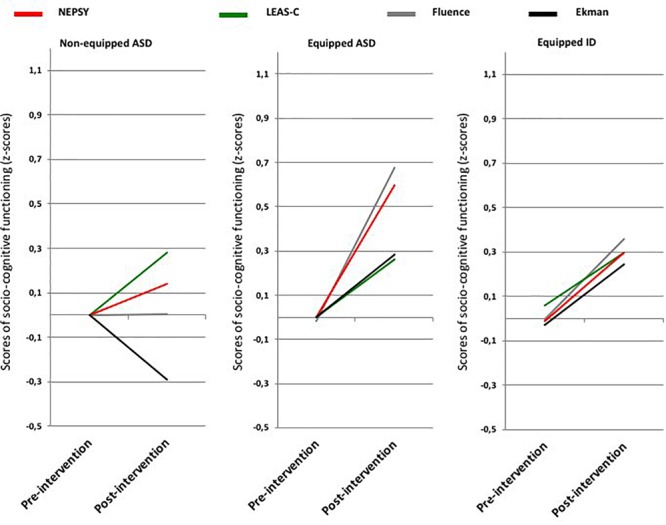
Pre- and post-intervention scores on each socio-cognitive test for each group (control-ASD vs. tablet-ASD vs. tablet-ID).

MANOVA revealed a simple interaction effect Time ^∗^ Group [*F*(2,45) = 3.78; *p* = 0.030; η^2^ = 0.144]. Partial MANOVAs revealed a significant effect for Time factor for tablet-ASD [*F*(1,13) = 30.87; *p* < 0.001; η^2^ = 0.704] and for tablet-ID [*F*(1,18) = 10.52; *p* = 0.005; η^2^ = 0.369]. No significant effect was reported for control-ASD. See Appendix Tables 3 and 4.

Thus, these results indicated that control-ASD did not significantly improve their performance across time. However, the two equipped groups exhibited significant improvements across time, with greater statistical effect for tablet-ASD compared with tablet-ID.

### Socio-Adaptive Behaviors in School Settings (EQCA-VS) (See Figure 5)

**FIGURE 5:**
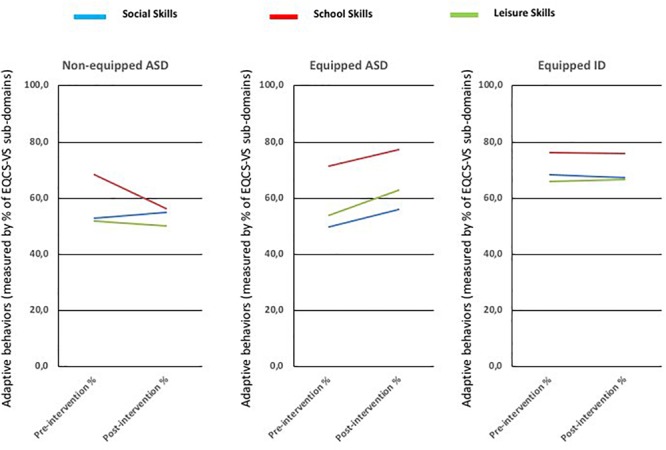
Pre- and post-intervention scores of Social, School, and Leisure behaviors of EQCA-VS for each group (control-ASD vs. tablet-ASD vs. tablet-ID).

MANOVA revealed a triple interaction effect Time ^∗^ Group ^∗^ EQCA-VS [*F*(8,180) = 3.20; *p* < 0.001; η^2^ = 0.066] indicating different results across time for each group and sub-domains of EQCA-VS. This result allowed us to conduct time comparisons analyses by sub-domain of EQCA-VS (See Appendix Tables 1, 3, and 3a). They revealed the following results:

#### Autonomy

Comparisons did not reveal any significant effect for any of the three groups.

#### Communication

Comparisons did not reveal any significant effect for any of the three groups.

#### Social Skills

The Time effect was only significant for tablet-TSA [*t*(13) = -2.35; *p* = 0.035]. No significant effect was obtained for the two other groups. It is to be noted that the performance of the control-TSA group was superior to the performance of the tablet-TSA group at the beginning of the intervention, whereas both were equivalent at the end of the 3-month intervention.

#### School Skills

A Time effect for tablet-TSA was observed [*t*(13) = -3.1; *p* = 0.008]. No significant effect was obtained for the two other groups.

#### Leisure

ANOVA revealed a Time effect for tablet-TSA [*t*(13) = -2.18; *p* = 0.049]. No significant effect was obtained for the two other groups.

Thus, only the group of tablet-ASD presents improved performance on post-intervention condition compared with the two other groups in three dimensions of EQCA-VS: Social skills, School skills, and Leisure (see Figure 5). Results of the three groups on Autonomy and Communication are presented in Table 3 of Appendix.

### Social Response in School Settings (SRS) (See Figure 6)

**FIGURE 6:**
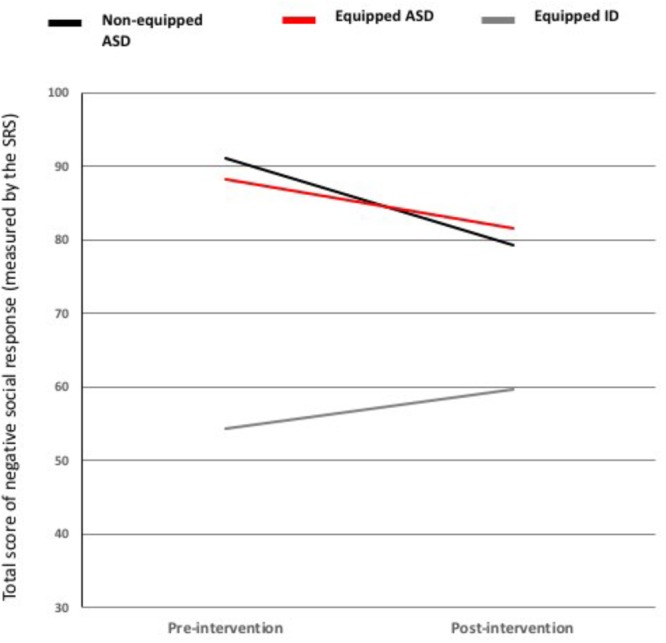
Pre- and post-intervention scores on each domain of SRS for each group (control-ASD vs. tablet-ASD vs. tablet-ID).

MANOVA revealed a significant interaction effects with two factors: Time ^∗^ Group [*F*(2,45) = 3.63; *p* = 0.034; η^2^ = 0.139]. Pairwise comparisons showed a significant decrease for both ASD groups (i.e., tablet and control) compared with ID [ID vs. tablet-ASD: *p* = 0.039, *SE* = 10.97; ID vs. control-ASD: *p* = 0.032, *SE* = 10.76]. This result allowed us to conduct time comparisons analyses by sub-domain of SRS. See Appendix Tables 4 and 4a.

#### Communication

Comparisons did not reveal any significant effect for any of the three groups.

#### Cognition

Comparisons did not reveal any significant effect for any of the three groups.

#### Awareness

Comparisons did not reveal any significant effect for any of the three groups.

#### Motivation

The Time effect was significant for both ASD groups [tablet-ASD: *t*(13) = 2.188; *p* = 0.047; control-ASD: *t*(14) = 2.988; *p* = 0.010].

#### Repetitive Behaviors

The Time effect was only significant for the tablet-TSA group [*t*(13) = 2.463; *p* = 0.029]. No significant effect was obtained for the other group.

Thus, both ASD groups exhibited increased motivation toward social behaviors at the end of the intervention. Moreover, only the tablet-ASD group significantly reduced their repetitive behaviors at the end of the intervention.

## Discussion

To the best of our knowledge, no experimental study deployed and validated a technology aimed to both the assistance and the cognitive rehabilitation of children with ASD for their inclusion in mainstream classrooms. This section discusses results presented above, reporting significant improvements in terms of socio-cognitive functioning, behavior adaptation, and social response in school settings of children with ASD who were equipped with “School+” applications.

### “School+”: A Relevant Intervention for Children With ASD in Mainstream School Settings

The results of our study suggest that reported benefits in terms of socio-cognitive functioning for both groups equipped with “School+,” with a greater impact for tablet-ASD compared with tablet-ID. At the end of intervention, tablet-ASD children significantly increased their performance on socio-cognitive measures, including face memory, facial emotions identifications, emotion lexicon and self and others’ emotional awareness. Our study also reveals benefits from using “School+” applications in terms of behavior adaptation of children with ASD are related to the use of “School+” in this environment. Indeed, at end of the 3-month intervention, only children with ASD who were equipped with “School+” applications significantly improved their behaviors, compared with control-ASD children. However, these improvements were partial and only concerned three domains of five ones measured by EQCA-VS scale, i.e., Social skills, School skills, and Leisure. This result is consistent with a preliminary study presenting improved task performance of five children with ASD (compared with five control) in mainstream classrooms when using a tablet-based activity schedule to assist classroom routines and verbal communication activities (Fage et al., 2014). Thus, the observation of enlarged benefits (Social skills, School skills, and Leisure, three of the five studied dimensions) indicates that a global and short intervention can be enough to efficiently reduce school disabilities of children with ASD. The association of compensatory *in situ* assistance, cognitive training on socio-cognitive processes, and executive functioning together allowed these benefits. As for the social response, measured by the SRS, both groups of children with ASD significantly improved their social response. This result could be related to the inclusion in mainstream classrooms. Many published works highlight the benefits of mainstream inclusion for students with ASD (Hunt and McDonnell, 2007).

#### Application Design Suited for Mainstream Classrooms

School aides reported that children autonomously used our applications at the end of the 3-month intervention in mainstream classrooms. Parents’ scores on the USE questionnaire show the high usability of the “School+” package. The reported high usability of our technological support in a daily life environment may be underpinned by the design principles that have been implemented through our approach. For example, the significant reductions of repetitive behaviors could be related to the idea that structured and predictable interfaces of applications reduces anxiety associated with mainstream environments for children with ASD (Hayes et al., 2010). More broadly, applying principles of successful cognitive behavioral interventions to the design of technological supports appeared to be suited for their usage in such stressful environments.

#### A Solution That Can Be Enriched

Nonetheless, it is to be noted that difficulties related to communication and autonomy, as measured by EQCA-VS, have not been significantly improved in “School+” intervention. Similarly, no significant decrease of Communication, Cognition, and Awareness has been observed, as assessed by SRS. Several explanations can possibly be formulated. First, the short intervention time (i.e., 3 months) could justify the absence of significant effects on complex situated behaviors as measured by these two questionnaires. Second, the spectrum of assistances and rehabilitations currently implemented in “School+” do not cover all the needs in terms of communication and autonomy in school settings. For instance, for rehabilitation applications, social stories (Nikopoulos and Keenan, 2007) or even problem solving (Sansosti and Powell-Smith, 2008) aimed toward school settings could allow wider benefits, especially on aforementioned domains.

### “School+”: Specific and Transversal Benefits Across Populations

The inclusion of a group of children with another condition (i.e., Intellectual Disabilities), also equipped with our technology, allowed us to enrich our results with two main elements. First, benefits from using “School+” are not reported for children with ID on either socio-adaptive behaviors (measured by EQCA-VS) or social response (measured by SRS). This suggests a specific efficacy of these applications for children with ASD. As such, these results confirm the work presented by Fage et al. (2016) where children with ID improved on school routines but not on communication routines. However, unlike this previous work, we now have a larger number of participants, strengthening the observed statistical effects. It is also to be noted that the absence of a significant decrease of the SRS dimensions for children with ID is consistent with the literature, since such behaviors, usually related to ASD, are less common among children with ID (Benson and Fuchs, 1999). Second, unlike other dimensions, children with ID exhibited significant improvements on measures of socio-cognitive mechanisms. More specifically, statistical size effects were stronger for children with ASD (η^2^ = 0.704) than for children with ID (η^2^ = 0.369). Simultaneously, children with ASD who did not follow “School+” training exhibited statistically equal performance across time. This result suggests that children with ID benefited from using “School+” applications in terms of socio-cognitive functioning.

Moreover, these different results are strengthened by interaction data indicating no statistical differences between the two equipped groups in terms of amount of uses of the “School+” applications during the intervention time. In other words, when using equally our assistive and rehabilitation applications, children with ASD increased their socio-adaptive behaviors and their social response, while children with ID exhibited no significant differences across time on these domains. However, both groups significantly increased their performance on socio-cognitive mechanisms.

### A Systematic Approach for Global Benefits

Benefits of systematic approaches for children with ASD have previously been highlighted in numerous studies (Ospina et al., 2008). Results of our intervention suggest that such approaches, usually implemented in specialized settings, can also be suited for mainstream environments (e.g., schools) thanks to the new opportunities provided by technological supports. As proposed in interventions such as Lovaas or TEACCH, “School+” relies on a close collaboration between families and school staffs to propose contents that are personalized to each child (Lovaas, 1987; Panerai et al., 2002).

Moreover, it is noteworthy that the two groups of children with ASD (i.e., tablet and control) significantly improved their behaviors toward social interactions at the end of the 3-month intervention in mainstream classrooms. Consistent with the literature, which emphasizes the benefits of inclusion in mainstream environments in terms of social participation of children with ASD (Hunt and McDonnell, 2007). This result urges researchers to do more work toward the inclusion of children with ASD in mainstream environments. Also, the fact that both groups of students with ASD improved on this measure urges for including a second control group with another condition (students with ID in the present study). In this case, a cross-syndrome design allowed us to isolate effects that are specific for our target population.

Finally, our study had positive effects on inclusion plans of participating schools. Our intervention allowed some children, who used to be identified by school staff as “misfit for inclusion in mainstream classrooms,” to benefit from such an inclusion with sometimes dramatic improvements of their socio-adaptive and autonomy behaviors. This situation resulted in increasing time spent in inclusion, and even attending new classes in mainstream classrooms for some participants.

### Limitations and Perspectives

Regarding experimentation duration, 1 h per week during 3 months represents a very short time to validate a *m*TBI intervention. Given this short time of intervention, reported benefits in terms of socio-adaptive behaviors and socio-cognitive functioning suggest that the “School+” solution is particularly relevant to support school inclusion of children with ASD. A longitudinal study, with evaluation after 6 months and after 9 months of use for instance, could strengthen these results and evaluate the durability of these effects (i.e., upholding of adaptive behaviors across time), as well as socio-cognitive functioning of tablet-ASD children (on socio-cognitive processes). Hence, the present results can be seen as promising for a pilot study performed with a short intervention duration.

Experience from each stakeholder involved in the project has been capitalized, as well as their suggestions to improve this kind of intervention. Hence, to further explore avenues opened by our approach, an interesting direction could be adding contents by creating new activity schedules to cover as many school setting tasks as possible. In the same vein, enrichment of rehabilitation applications contents could allow to obtain greater benefits in terms of socio-cognitive functioning.

## Conclusion

This study presents an intervention that relies on a set of mobile applications, “School+,” to support school inclusion of children with ASD in mainstream classrooms. These assistive and rehabilitation applications have been used for 3 months by 33 children (14 children with ASD and 19 children with ID) from special-education classrooms, during their first inclusion in mainstream classrooms in secondary schools. Fifteen children with ASD, who were not equipped with the applications, also participated to our study as a comparative group; the group of tablet-ID children allowed us to verify whether observed benefits were specific to the target population (ASD) or common with others (ID for instance).

Tablet-ASD children exhibited improvements on socio-cognitive functioning (as assessed by four neuropsychological tests), three domains of socio-adaptive behaviors (Social skills, School skills, and Leisure), and two domains of social response (motivation and repetitive behaviors). Tablet-ID children also exhibited improved performance on these tests at the end of the intervention.

Thanks to a systematic approach, based both on *in situ* assistance and cognitive training of socio-cognitive processes, the 3-month intervention based on “School+” applications allowed participants with ASD to be more included in mainstream classrooms for better social participation. Taken together, these results are promising and support the inclusion of *m*TBI in therapeutic and compensatory ecological interventions for children with ASD.

## Author Contributions

CF developed and implemented the intervention, collected the data, conducted the statistical analyses, and wrote the manuscript. CC and EB participated in developing the intervention and contacting school settings for the intervention implementation and gave comments to improve the manuscript. KE, AA, and MB conducted TSA diagnoses, helped recruit participants, and provided insights to improve results discussion. HS developed and helped implementing the intervention, and contributed on the writing to improve the manuscript.

## Conflict of Interest Statement

The authors declare that the research was conducted in the absence of any commercial or financial relationships that could be construed as a potential conflict of interest. The reviewer AC and handling Editor declared their shared affiliation at time of review.

## Footnotes

1 According to Sigman and Ruskin (1999), if syndrome group A and contrast group B are matched on chronological age and intellectual functioning, but the mean of group A on an intervention effect is significantly higher than the mean of group B, then group A is considered to exhibit a specific benefit of the intervention. A benefit (or pattern of benefit) is considered unique to syndrome A if it is evidenced only by individuals who have this syndrome. Another advantage of the cross-syndrome design is that it allows single-blind evaluations of the participants: the evaluator does not know in which group the child will be until neuro-pediatricians delivered their diagnosis, at the data analysis step. This process ensures all participants are evaluated in the exact same way, regardless of their condition.
