# Supplementary material for: Tablet Apps to Support First School Inclusion of Children With Autism Spectrum Disorders (ASD) in Mainstream Classrooms: A Pilot Study
Source: Front Psychol. 2018 Oct 23;9:2020. doi: 10.3389/fpsyg.2018.02020 (PMC6207048; doi:10.3389/fpsyg.2018.02020)
Supplement: Supplementary file 1 [file Table_1.docx]

**Supplementary material**

|  | **Non-equipped ASD** | | **Equipped ASD** | | **Equipped ID** | |
| --- | --- | --- | --- | --- | --- | --- |
|  | Pre | Post | Pre | Post | Pre | Post |
| **Autonomy** M  (SD) | 17.20  (7.78) | 19.00  (1.54) | 20.71  (2.00) | 21.07  (9.60) | 22.84  (1.98) | 24.05  (7.90) |
| **Communication** M  (SD) | 21.60  (2.44) | 21.80  (1.93) | 20.07  (2.90) | 20.14  (2.87) | 23.84  (2.08) | 23.84  (2.08) |

Table 1. Pre and Post intervention scores of Autonomy and Communication behaviors EQCA-VS for each group (Non-equipped ASD vs. Equipped ASD vs. Equipped ID).

| Measured Ability | Sub-domain | Time | Non-equipped ASD | Equipped ASD | Equipped ID |
| --- | --- | --- | --- | --- | --- |
| Adaptive school behaviors | Communication | Pre-intervention | M=21.60  σ=9.44 | M=20.07  σ=10.86 | M=23.84  σ=9.08 |
|  |  | Post-intervention | M=21.80  σ=7.47 | M=20.14  σ=10.73 | M=23.84  σ=9.08 |
|  | Social skills | Pre-intervention | M=18.00  σ=8.85 | M=16.93  σ=10.22 | M=23.21  σ=9.47 |
|  |  | Post-intervention | M=18.73  σ=7.53 | M=19.00  σ=11.24 | M=24.68  σ=9.72 |
|  | Autonomy | Pre-intervention | M=17.20  σ=7.78 | M=20.71  σ=7.49 | M=22.84  σ=8.61 |
|  |  | Post-intervention | M=19.00  σ=5.96 | M=21.07  σ=9.60 | M=24.05  σ=7.90 |
|  | School skills | Pre-intervention | M=34.20  σ=14.40 | M=35.71  σ=8.82 | M=38.05  σ=11.16 |
|  |  | Post-intervention | M=28.20  σ=12.98 | M=38.64  σ=10.32 | M=37.89  σ=10.79 |
|  | Leisure | Pre-intervention | M=11.40  σ=6.19 | M=11.86  σ=5.45 | M=14.47  σ=6.70 |
|  |  | Post-intervention | M=11.00  σ=3.82 | M=13.79  σ=6.75 | M=14.68  σ=6.66 |
| Social Response | Communication | Pre-intervention | M=30.93  σ=13.18 | M=30.00  σ=12.84 | M=20.05  σ=10.05 |
|  |  | Post-intervention | M=25.73  σ=8.79 | M=28.14  σ=12.38 | M=21.42  σ=11.15 |
|  | Cognition | Pre-intervention | M=17.47  σ=8.15 | M=16.07  σ=8.65 | M=10.00  σ=7.67 |
|  |  | Post-intervention | M=15.93  σ=5.80 | M=16.93  σ=9.19 | M=11.74  σ=6.95 |
|  | Awareness | Pre-intervention | M=9.80  σ=5.00 | M=10.86  σ=5.63 | M=5.84  σ=4.18 |
|  |  | Post-intervention | M=10.80  σ=2.81 | M=10.79  σ=5.29 | M=7.68  σ=4.18 |
|  | Motivation | Pre-intervention | M=16.73  σ=6.47 | M=13.43  σ=4.82 | M=8.89  σ=5.35 |
|  |  | Post-intervention | M=12.87  σ=4.24 | M=12.43  σ=5.14 | M=9.74  σ=5.59 |
|  | Repetitive behaviors | Pre-intervention | M=16.13  σ=6.83 | M=19.93  σ=14.04 | M=9.53  σ=7.78 |
|  |  | Post-intervention | M=13.00  σ=7.58 | M=14.43  σ=10.11 | M=9.16  σ=6.97 |
| Socio-cognitive Functioning | Emotional Fluency | Pre-intervention | M=.00  σ=1.00 | M=-.01  σ=1.03 | M=.01  σ=1.02 |
|  |  | Post-intervention | M=.00  σ=.89 | M=.68  σ=1.03 | M=.38  σ=.82 |
|  | Emotional Awareness | Pre-intervention | M=.00  σ=1.00 | M=.00  σ=.96 | M=-.05  σ=1.00 |
|  |  | Post-intervention | M=.28  σ=.68 | M=.26  σ=1.25 | M=.28  σ=.86 |
|  | Faces Memory | Pre-intervention | M=.00  σ=1.00 | M=.00  σ=.96 | M=-.00  σ=.97 |
|  |  | Post-intervention | M=.14  σ=.87 | M=.60  σ=.90 | M=.36  σ=.98 |
|  | Facial Emotion Identification | Pre-intervention | M=.00  σ=1.00 | M=.00  σ=.96 | M=-.05  σ=1.00 |
|  |  | Post-intervention | M=-.29  σ=.90 | M=.28  σ=.82 | M=.25  σ=1.03 |

Table 2. Means and Standard Deviations on all three measured abilities.

| Measured Ability | Global MANOVA | | | | | | Student t test |
| --- | --- | --- | --- | --- | --- | --- | --- |
|  | ***Time Effect*** | ***Measure Effect*** | ***Time* x *Group interaction*** | ***Measure* x *Group* *interaction*** | ***Time* x *Measure interaction*** | ***Time* x *Measure* x *Group interaction*** |  |
| Adaptive School beha  viors | F(1,45)=.83  p=.369  η^2^=.018 | F(4,180)=152.78  p<.001  η^2^=.772 | F(2,45)=1.70  p=.194  η^2^=.070 | F(8,180)=1.94  p=.056  η^2^=.079 | F(4,180)=3.20  p=.014  η^2^=.066 | F(8,180)=4.34  p<.001  η^2^=.162 | See Tab 3a |
| Social Response | F(1,45)=2.80  p=.101  η^2^=.059 | F(4,180)=104  p<.001  η^2^=.698 | F(2,45)=3.63  p=.034  η^2^=.139 | F(4,180)=1.34  p=.228  η^2^=.056 | F(4,180)=6.26  p<.001  η^2^=.122 | F(8,180)=1.81  p=.078  η^2^=.074 | - |
| Socio-cognitive Functioning | F(1,45)=19.06  p<.001  η^2^=.298 | F(3,135)=.68  p=.567  η^2^=.015 | F(2,45)=3.78  p=.030  η^2^=.144 | F(6,135)=.30  p=.935  η^2^=.013 | F(3,135)=1.43  p=.237  η^2^=.031 | F(6,135)=.93  p=.478  η^2^=.040 | - |

Table 3. Global MANOVA effects on all three measured abilities.

| ^EQCA Sub-domain^ | ^Non-equipped ASD^ | ^Equipped ASD^ | ^Equipped ID^ |
| --- | --- | --- | --- |
| ^Communication^ | t(14)=-.146  p=.886 | t(13)=-1.00  p=.336 | t(18)=  p= |
| ^Social skills^ | t(14)=-.416  p=.684 | t(13)=-2.354  p=.035 | t(18)=-1.527  p=.144 |
| ^Autonomy^ | t(14)=-1.304  p=.213 | t(13)=-.324  p=.751 | t(18)=-1.985  p=.063 |
| ^School skills^ | t(14)=2.064  p=.058 | t(13)=-3.114  p=.008 | t(18)=.232  p=.819 |
| ^Leisure^ | t(14)=.282  p=.782 | t(13)=-2.176  p=.049 | t(18)=-.809  p=.429 |

Table 3 a. Student t test effects for sub-domains of Adaptive School Behaviors measure (EQCA).

| Measured Ability | Group | Partial MANOVA | | | | Student t test |
| --- | --- | --- | --- | --- | --- | --- |
|  |  | ***Time Effect*** | ***Measure Effect*** | ***Time x Measure* *Interaction*** |  | |
| Social Response  (SRS) | Non-equipped ASD | F(1,14)=3.20  p=.095  η^2^=.186 | F(4,56)=44.10  p<.001  η^2^=.759 | F(4,56)=3.56  p=.012  η^2^=.203 | See Tab 4a | |
|  | Equipped ASD | F(1,13)=2.11  p=.170  η^2^=.139 | F(4,52)=28.06  p<.001  η^2^=.683 | F(4,52)=2.82  p=.034  η^2^=.178 | See Tab 4a | |
|  | Equipped ID | F(1,18)=3.30  p=.086  η^2^=.155 | F(4,72)=34.78  p<.001  η^2^=.659 | F(4,72)=1.68  p=.165  η^2^=.085 | - | |
| Socio-cognitive Functioning  (Neuro-psychological tests) | Non-equipped ASD | F(1,14)=.06  p=.808  η^2^=.004 | F(3,42)=.83  p=.486  η^2^=.056 | F(3,42)=1.77  p=.168  η^2^=.112 | - | |
|  | Equipped ASD | F(1,13)=30.89  p<.001  η^2^=.704 | F(3,39)=.54  p=.655  η^2^=.040 | F(3,39)=1.35  p=.272  η^2^=.094 | - | |
|  | Equipped ID | F(1,18)=10.52  p=.005  η^2^=.369 | F(3,54)=.10  p=.959  η^2^=.006 | F(3,54)=.04  p=.989  η^2^=.002 | - | |
|  |  |  |  |  |  | |

Table 4. Partial MANOVA effects for Social Response and Socio-cognitive functioning measures.

| ^SRS Sub-domain^ | ^Non-equipped ASD^ | ^Equipped ASD^ |
| --- | --- | --- |
| ^Communication^ | t(14)=1.809  p=.092 | t(13)=.809  p=.433 |
| ^Cognition^ | t(14)=1.06  p=.307 | t(13)=-5.545  p=.595 |
| ^Awareness^ | t(14)=-.885  p=.391 | t(13)=.072  p=.944 |
| ^Motivation^ | t(14)=2.988  p=.010 | t(13)=2.188  p=.047 |
| ^Repetitive behaviors^ | t(14)=1.752  p=.102 | t(13)=2.463  p=.029 |

Table 4 a. Student t test effects for Sub-domains of Social Response measure (SRS).
